# CT diagnosis and endovascular management of spontaneous rupture of uterine artery in pregnancy

**DOI:** 10.1259/bjrcr.20150351

**Published:** 2016-11-11

**Authors:** Karthikeyan Damodharan

**Affiliations:** Department of Diagnostic Radiology, Singapore General Hospital, Singapore, Singapore

## Abstract

Spontaneous rupture of the uterine artery during pregnancy is an extremely uncommon condition, which presents as an acute emergency, associated with high rates of maternal and foetal mortality. This condition is challenging to diagnose and treat, as it is an uncommon entity having an acute nature of presentation. They are usually managed by emergency laparotomy and the diagnosis is rarely made preoperatively. We describe a case with CT angiographic images clearly demonstrating the uterine artery pseudoaneurysm with associated extensive haemoperitoneum, which helped us confirm this rare diagnosis. We successfully treated the patient by an endovascular approach, which has not been reported previously.

## Summary

Spontaneous rupture of the uterine artery during pregnancy is an extremely uncommon condition that presents as a clinical emergency and is associated with high rates of maternal and foetal mortality. This condition is challenging to diagnose and treat, as it is an uncommon entity that has an acute presentation with sudden onset of abdominal pain and circulatory collapse. It is usually managed by emergency laparotomy and the diagnosis is rarely made preoperatively. We describe a case in which CT angiographic images clearly demonstrated the uterine artery pseudoaneurysm with associated extensive haemoperitoneum, which helped us confirm this rare diagnosis. We successfully treated the patient through an endovascular approach, which has not been reported previously.

A 30-year-old primigravida with no significant past history of medical, surgical or endovascular interventions, presented at 34 weeks of gestation with a history of waking up with sudden onset of stabbing abdominal pain that was not suggestive of labour pain, vomiting and feeling faint. The patient reported that she could not perceive any foetal movements, but denied any history of recent trauma or per vaginal bleeding. She was hypotensive (blood pressure 70/40 mmHg) with a normal heart rate and was immediately fluid resuscitated.

On examination, the patient had a diffusely tender abdomen with cold, clammy peripheries. The obstetric assessment showed no signs of labour, closed cervix and absent foetal heart sounds. An artificial rupture of membranes was performed that showed clear liquid. Bedside obstetric ultrasound performed in the emergency room confirmed the absence of foetal heart activity and movements. A provisional diagnosis of placental abruption with foetal demise was made. The stillborn baby and placenta were delivered vaginally 6 h later.

A few hours postpartum, the patient complained of severe abdominal pain similar to the pain earlier in the day. The patient was noted to become hypotensive and tachycardic, with generalized abdominal distension and tenderness. Full blood count analysis revealed a significant drop in haemoglobin and platelets but normal coagulation profile.

The patient was transferred to the high dependency care unit and was transfused three units of packed red cells and two units of platelets. Bedside ultrasound was performed that showed free fluid around the liver. Spontaneous rupture of the liver was suspected and a non-contrast CT scan of the abdomen was performed that showed haemorrhagic fluid around the liver, which was intact. Clinically, the patient continued to show signs of bleeding over the next few hours, with low haemoglobin levels on repeat analysis despite the transfusion. However, she remained haemodynamically stable with mild tachycardia.

**Figure 1.**
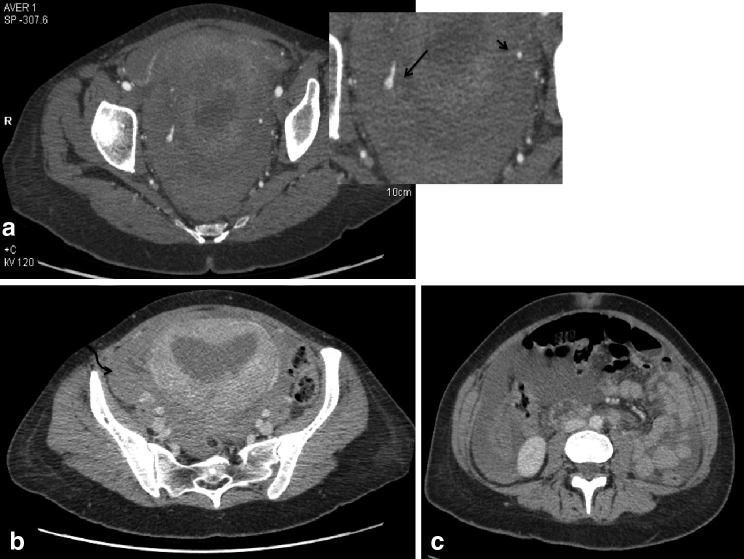
(a) Axial image from the CT angiogram of the pelvis demonstrates right uterine artery pseudoaneurysm indicated by the focal abnormal dilatation of the artery (long arrow in the enlarged inset image) compared with the normal calibre left uterine artery (short arrow). (b) Delayed venous phase axial image of the pelvis demonstrating enhancing enlarged postpartum uterus and high-density fluid in the pelvis (curved arrow). (c) Haemoperitoneum within the abdomen.

**Figure 2.**
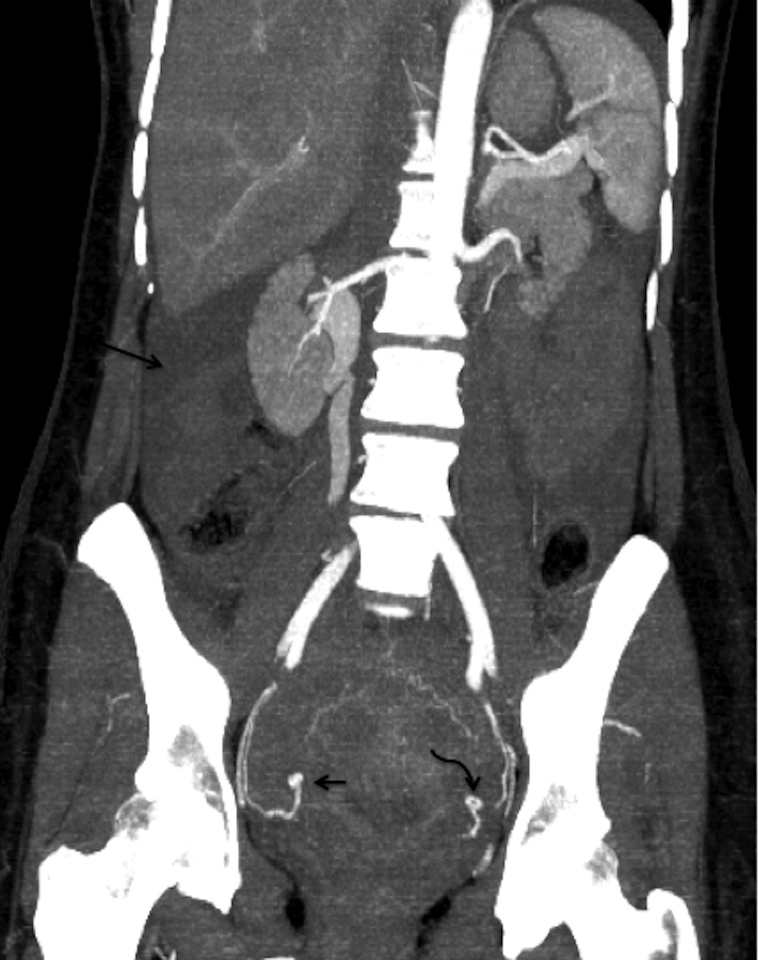
Coronal maximum intensity projection reconstructed images of the CT angiogram clearly demonstrates the pseudoaneurysm of the ascending portion of the right uterine artery (short arrow) compared with normal appearance of the left uterine artery (curved arrow). Long arrow indicates free fluid in the abdomen on the right side.

**Figure 3.**
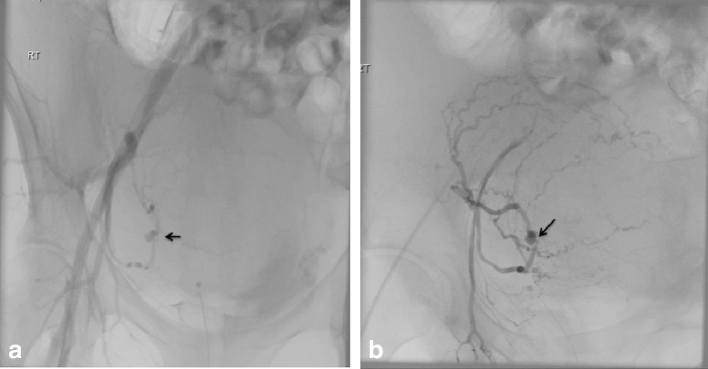
(a, b) Conventional pelvic angiogram images obtained by contrast injection via catheters placed in the right common iliac and right internal iliac artery, respectively. Both demonstrate the right uterine artery pseudoaneurysm (arrows) as seen on the CT angiogram images (Figure 2). Also shown is increased vascularity of the postpartum uterus.

**Figure 4.**
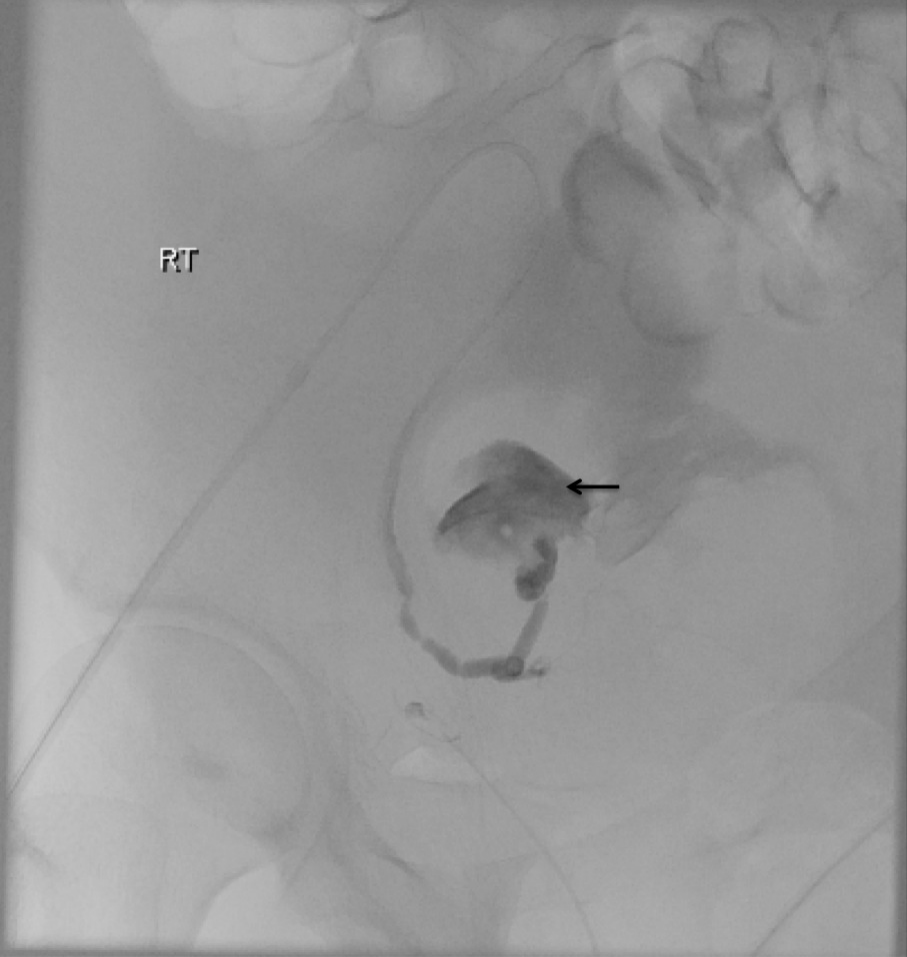
Selective angiogram of the right uterine artery with the catheter tip in the proximal right uterine artery clearly shows the pseudoaneurysm with active contrast extravasation (arrow) indicative of active bleed.

**Figure 5.**
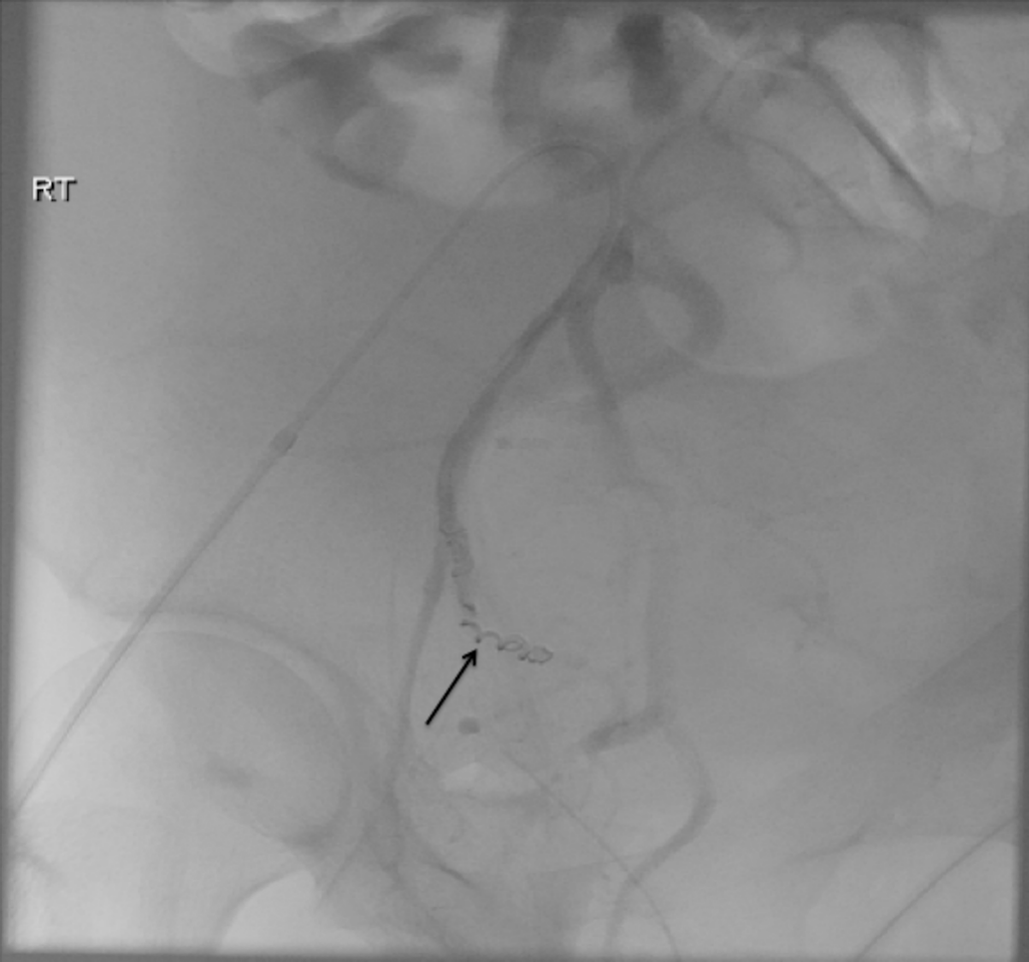
Selective angiogram of the right uterine artery after coil (arrow) embolization of the mid portion of the vessel shows no filling of the previously seen pseudoaneurysm or active bleed.

The obstetric team sought the advice of the on-call interventional radiologist to explore the possibility of embolization in view of the suspected diagnosis of ruptured liver. A CT angiogram of the abdomen and pelvis was advised as the initial investigation to localize the bleeding site, as the patient remained clinically stable. This revealed a focal dilatation of the ascending segment of the right uterine artery measuring 6 mm, suggestive of a pseudoaneurysm and extensive haemoperitoneum within the abdomen and pelvis (Figures 1 and 2). Following discussion between the gynaecologists and the interventional radiologist, the patient was transferred immediately within the next hour to the interventional suite. A pelvic angiogram was performed followed by selective bilateral uterine arteriograms. A right uterine artery pseudoaneurysm was demonstrated on the right iliac angiogram (Figure 3) corresponding to the CT angiogram findings with no active bleeding. A selective right uterine artery angiogram demonstrated active contrast extravasation, which was probably owing to the disruption of the clot surrounding the pseudoaneurysm due to the pressure from the contrast injection directly into the mid-uterine artery as opposed to the non-selective earlier angiograms (Figure 4). Immediate embolization of the uterine artery proximal to the pseudoaneurysm was performed using fibred platinum coils (035" 5 mm × 4.5 mm VortX^™^ 35, Boston Scientific Corporation, Hemel Hempstead, UK). The final angiogram showed no evidence of the previously demonstrated pseudoaneurysm or bleeding and stasis of flow in the right uterine artery (Figure 5). Subsequently, the patient remained haemodynamically stable and asymptomatic with no evidence of further bleeding and was transferred back to the maternity unit for grievance counselling. Histology of the placenta confirmed an intact placenta with no evidence of abruption and findings compatible with foetal demise within 24 h prior to delivery. The patient remained asymptomatic and well at 3-month outpatient follow-up.

## Discussion

Spontaneous rupture of the uterine artery in pregnancy with haemoperitoneum is a very rare cause of acute onset of severe abdominal pain and maternal hypovolaemic shock during pregnancy. Our recent Medline literature search revealed that fewer than 10 cases of this rare condition have been reported so far.^1–4^ Nevertheless, as radiologists, it is essential to be aware of this diagnosis in pregnant females presenting with acute onset of unexplained abdominal pain with a significant drop in blood haemoglobin, to aid prompt identification and management. Some patients do not develop profound haemodynamic instability^3^ as observed in our patient. This is likely owing to the pressure effect of the gravid uterus on the uterine artery, as well as the ability of these young patients to cope with decreasing blood volume^3^. Our patient probably had an initial episode of significant bleeding at the time of presentation, which may have caused significant compromise of the foetal circulation and demise. Following this, the patient continued to have intermittent episodic bleeding, but remained haemodynamically stable, probably owing to ongoing resuscitation in addition to the factors mentioned above.

Spontaneous haemoperitoneum in the third trimester of pregnancy could be either due to rupture of the uterine arteries or utero-ovarian veins.^1^ Maternal mortality rate from this condition has been reported as 3.6% in the recent literature compared with 49.6% in the earlier series, which could be attributed to the advances in the resuscitative and operative techniques.^5^ Placental abruption is the most common initial diagnosis made in patients presenting with spontaneous rupture of uterine arteries, and significant drop in haemoglobin levels is a common finding,^2^ as observed in our patient. Other differential diagnoses include spontaneous rupture of the liver, ruptured uterus, abdominal pregnancy, perforated appendicitis and rupture of visceral artery aneurysms.^1,5^

These patients are treated with prompt resuscitation, blood product transfusion and emergency laparotomy, and ligation of the ruptured uterine artery,^2,3^ as most patients present with dramatic haemodynamic collapse that leaves little scope for imaging investigations and the diagnosis is usually made at laparotomy.^5^

A significant drop in the haemoglobin levels is a common finding in these patients.^5^ Bedside ultrasound can be useful in identifying free intra-abdominal fluid/haematoma as well as the condition of the foetus.^1^

Our patient presented with haemodynamic instability that responded well to fluid resuscitation/blood product replacement and had subsequent episodic bleeding. This allowed us the opportunity to perform a diagnostic CT angiogram that confirmed the diagnosis, thereby enabling us to target and embolize the bleeding vessel. Coil embolization of the uterine artery pseudoaneurysm is already a well-established endovascular technique in the management of postpartum haemorrhage,^6^ which can be easily applied in this condition. This helped our patient avoid a major laparotomy with its own inherent risks of morbidity and mortality in the setting of haemodynamic instability and active blood loss.

We report a case of spontaneous uterine artery rupture, where the diagnosis was made using CT angiogram, which demonstrating the pseudoaneurysm, and subsequently treated successfully with a minimally invasive endovascular approach, in contrast to the prior reported cases requiring surgical treatment. Owing to the current advances in resuscitation techniques and the immediate availability of CT scanners with rapid image acquisition capabilities in most hospitals, in future, it may be possible to diagnose more such patients by imaging and treat them by embolization. Hence it is essential for radiologists to be aware of this rare condition, its imaging findings and endovascular treatment option to reduce the risks of major surgery in these patients.

## Learning points

1. Spontaneous uterine artery rupture during pregnancy is an extremely uncommon condition, presenting with acute onset of abdominal pain and hypotension.
2. Differential diagnosis includes placental abruption, ruptured uterus and spontaneous rupture of liver/spleen, or visceral artery aneurysms.
3. Ultrasound usually shows free intraperitoneal fluid/haematoma and signs of foetal distress/demise and can exclude placental abruption.
4. CT angiogram is readily available and can quickly confirm the diagnosis, as well as help in planning the embolization procedure by localizing the bleeding vessel.

## Consent

Written informed consent was obtained from the patient for publication of this case report, including the accompanying images.
